# Dr. Richard J. Levis

**Published:** 1890-12

**Authors:** 


Dr. Richard J. Levis, of Philadelphia, died of pneumonia at his suburban residence, "Cedarcroft," November 12, 1890, aged 63 years. Dr. Levis had been a conspicuous member of the profession for so many years that it will be readily understood how deep is the mourning and how general the sorrow over the death of such a man. Without disparagement to others, it may be said of Dr.

Levis that he was personally the most popular member of the profession in Philadelphia. He has been widely distinguished as a surgeon, and has occupied various places of honor which the partiality of his colleagues and not his ambition, secured to him.

